# The Adulteration of Drugs

**Published:** 1879-05

**Authors:** 


### The Adulteration of Drugs.

"Several mills in New England, and probably many elsewhere, are now engaged in grinding white stone into a fine powder, for purposes of adulteration. At some of these mills they grind three grades—soda grade, sugar grade, and flour grade. I am told that thousands of tons of it have been ground in one town of Massachusetts. It sells for about half a cent a pound. Flour has been adulterated in England, and probably here, with plaster-of-Paris, bone dust, sand, clay, chalk, and other articles. I am told that large quantities of damaged and unwholesome grain is ground in with flour, particularly with that kind called Graham flour. (To detect adulterations of flour, see *Sanitarian*, November, 1877.) Certainly hundreds, and probably thousands of barrels of 'terra alba,' or white earth, are sold in our cities every year, to be mixed with sugars in confectionery and other white substances. I am told by an eminent physician that this tends to produce stone, kidney complaints, and various diseases of the stomach. A Boston chemist tells me that he has found seventy-five per cent. of 'terra alba' in what was sold as cream of tartar used for cooking. A large New York house sells three grades of cream of tartar. A Boston chemist recently analyzed a sample of the best grade, and found fifty per cent. of 'terra alba' in that. Much of our confectionery contains thirty-three per cent. or more of 'terra alba.' The coloring matter of confectionery frequently contains lead, mercury, arsenic, and copper. Baking powders are widely sold which contain a large percentage of 'terra alba' and alum.—*Med. and Sur. Reporter.*

